# Supplementary material for: Conditional chemoconnectomics (cCCTomics) as a strategy for efficient and conditional targeting of chemical transmission
Source: eLife. 2024 Apr 30;12:RP91927. doi: 10.7554/eLife.91927 (PMC11060718; doi:10.7554/eLife.91927)
Supplement: Figure 3—source data 3. [file elife-91927-fig3-data3.zip › Figure 3 source data3 part 2.zip!/Figure 3-Source data 3-part2/1062-13-CRISPResso_Report_jt28fhr9.zip!/CRISPResso_on_1062_14/4d.gcaggaaatgagctacctac.Position_dependent_average_indel_size.pdf]

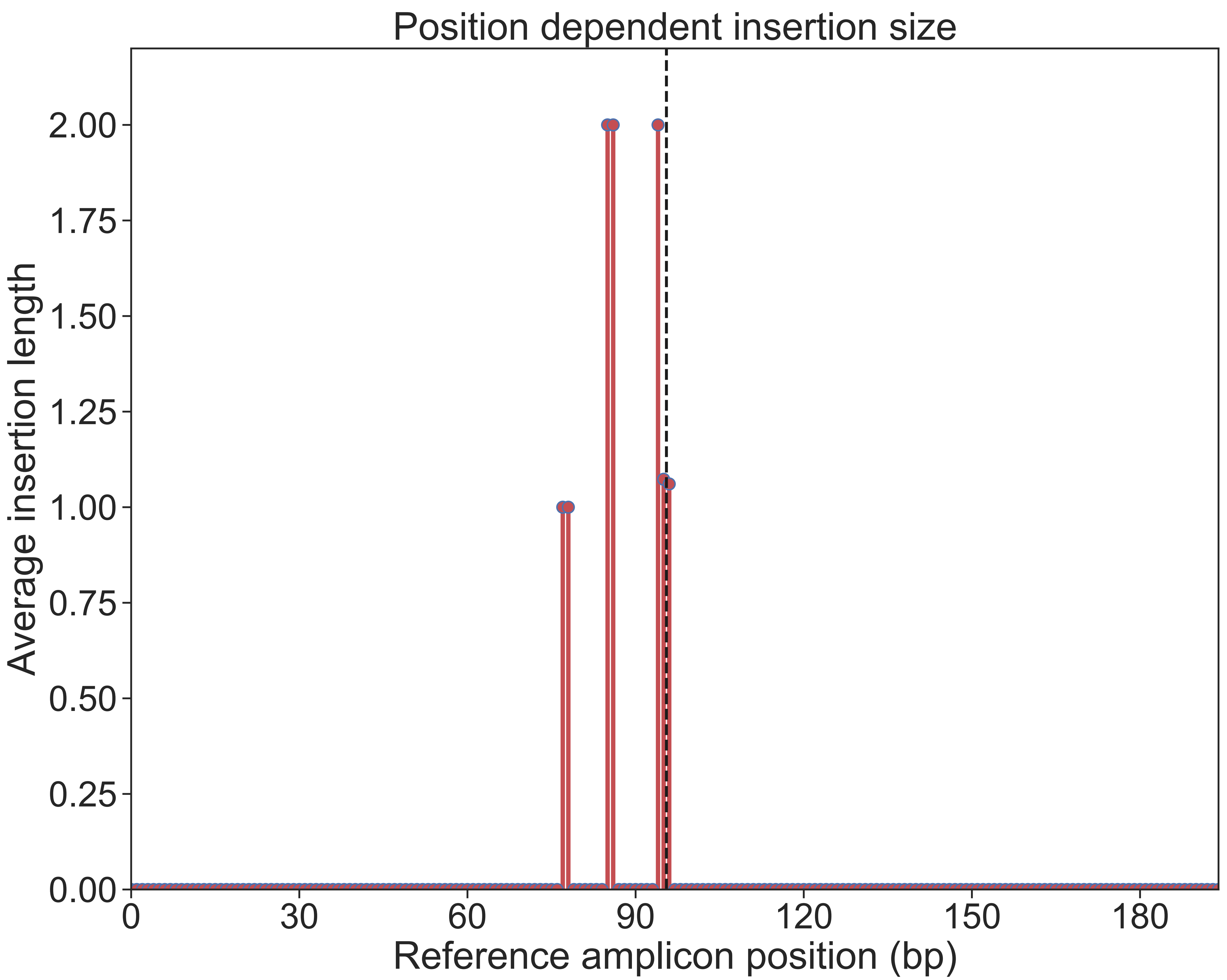
Position dependent insertion size
Average insertion length
0.00
0.25
0.50
0.75
1.00
1.25
1.50
1.75
2.00
0
30
60
90
120
150
180
Reference amplicon position (bp)

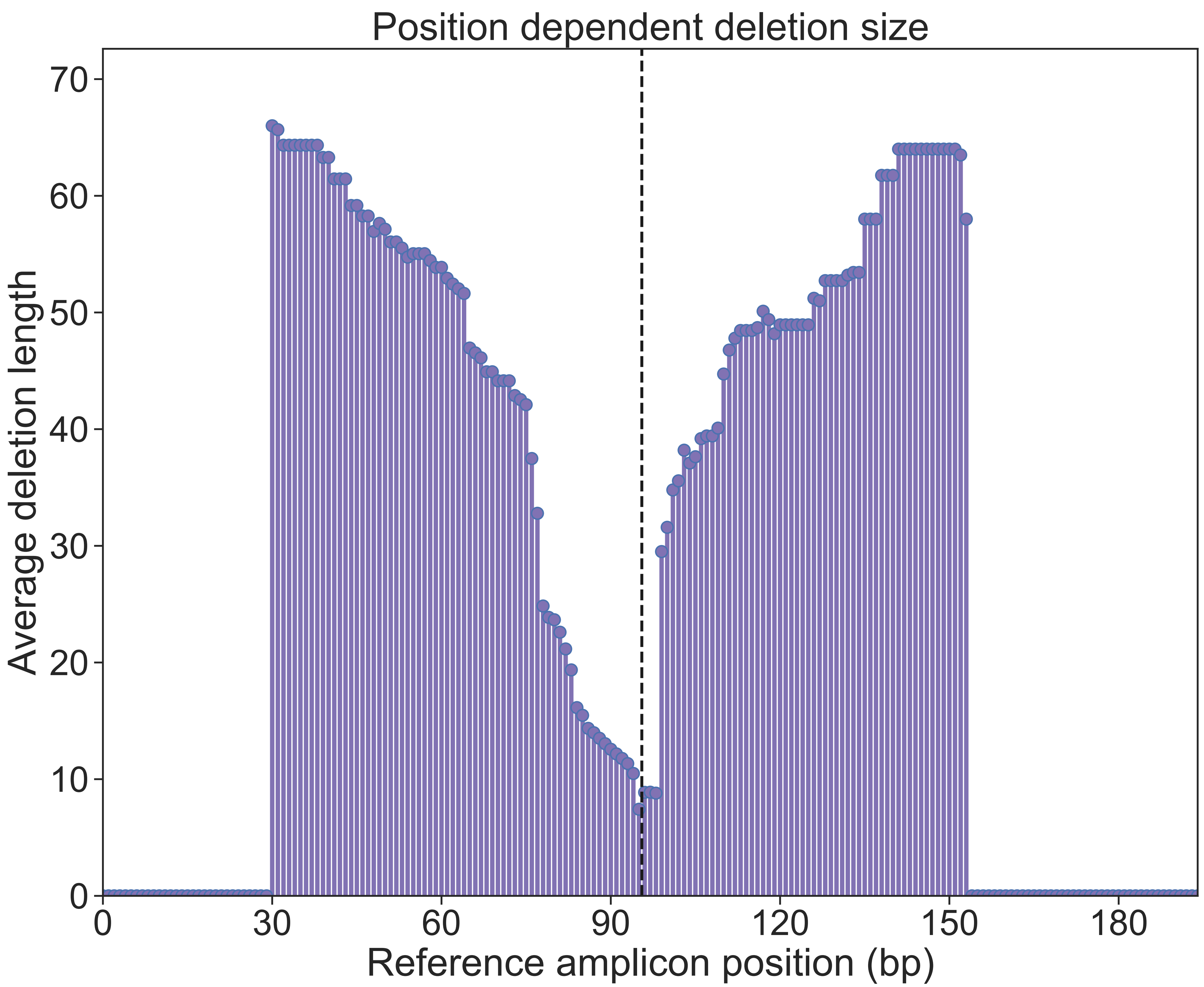
Position dependent deletion size
Average deletion length
0
10
20
30
40
50
60
70
0
30
60
90
120
150
180
Reference amplicon position (bp)
